# A Visit to the near East

**Published:** 1927

**Authors:** George Parker

**Affiliations:** Consulting Physician, Bristol General Hospital


### A VISIT TO THE NEAR EAST.

BY

George Parker, M.D. Cantab.,
*Consulting Physician, Bristol General Hospital.*

---

I have been asked to put down a few notes on my experiences in Palestine, whilst I was in medical charge of Sir Flinders Petrie's expedition south of Gaza for part of the season, and afterwards on a short holiday tour around some of the historic shrines.

There are at least three routes to Palestine : (1) the long sea passage by Gibraltar to Port Said and then by rail to Gaza and Jerusalem ; (2) the overland route by Marseilles or Brindisi and then by boat to Port Said ; (3) by Marseilles, but then taking a northern course by Athens, Smyrna, Cyprus to Beirut or Jaffa. This route gives one the charming scenery of the Greek Islands, Asia Minor and Syria. Some ships go alternately by routes (2) and (3). I met a man, indeed, who was trying a fourth way by the Turkish Railway through Asia Minor, and then from Constantinople by the Orient Express, which is practically overland all the way.

Cairo, where I had arranged to meet my party, is not only rich in ancient mosques, churches and bazaars, but it has also very fine modern streets and suburbs. Outside the town one sees the pyramids of Gizeh, and the Nile flowing through fields of sugar cane, cotton and maize, everywhere surrounded by limitless desert. Inside the streets one is struck with the brilliant colours of the men's dress. Here at last the sex is set free to adorn itself as other male animals do. A few men wear European clothes, capped by rich red tarbooshes, but the majority dress in bright blue, pink, or black gowns with an under-robe of yellow, white or striped silk, and above all this a turban of varied hues. In the museum one saw the really gorgeous relics of Tutankhamen's tomb, the jewels and carved work and golden busts. I was taken over the Kalaoun Hospital and then the El Azhar University, shown their treasures and MSS., and entertained most courteously by the Director, with sweet Persian tea, in his private divan.

The Palestine railroad landed me at 4 a.m. one December morning alone at Gaza, and I soon found myself riding a donkey, under the bright starlight, into the town, where our party was staying in an old Turkish mansion, used as a Government rest-house. While the mud huts were being built for our camp at Tell Gemmi in the desert, we filled up the time by unearthing some of the ancient Philistine walls of the town, possibly those which surrounded it when Samson carried off the gates, and in searching for the mound which Alexander the Great threw up in his siege of the city.

Gaza, among its gardens and cactus hedges, is still largely in ruins from Turkish destruction for materials, and from British shell fire. Our advance from Egypt to Jerusalem cost us 10,000 lives, and outside the town is a huge, well-kept British cemetery. The chief mosque, which is being beautifully restored, was used by the Turks to store their ammunition in during the war, till a British shell exploded it with sad results. It was once a fine Crusaders' church, built on the site of the ancient pagan temple. Though no longer a great centre of trade, the quaint little bazaars are thronged with purchasers. The streets are noisy, not only from the cries of the dealers, and from the camels and donkeys, but also from the many motor-cars sounding their horns, while above it all, especially at night, is the chant of the muezzin intoning the call to prayer.

The British Government is much in evidence. One sees a health office and dispensary, two good but small hospitals, one of them the property of the C.M.S., post and telephone offices, police headquarters, a town council house, primary and secondary schools, and a law court where the Chief Justice, Sir Thomas Haycraft, came to hold the assizes just as we were leaving. Everything has to be done economically in this poor country, wasted by war and previous neglect. However, the administration has succeeded wonderfully without any help from the British exchequer, and the country pays its way.

Blue books show that the British officials in Palestine only number 341, including railway and police officers. The Government has organised a legal code and an efficient judicial system. It is building up a register of all land titles. It has registered and given protection to all ancient buildings, *i.e.* all erected before 1700 A.D. It has created a fine police force of 1,100 men with British officers. It has taken over the railroads, brought them up to date, and put in order 600 miles of public roads, over which the native has promptly set up cheap motor services. The Education Department has already 314 government schools and 400 private ones with an attendance roll of 70,000, besides organising two training colleges and a higher examination of matriculation standard. The Health Department, with aid from the Rockefeller and Rothschild funds, is rapidly wiping out the great scourges of the country, malaria, rabies and trachoma. It provides trained nurses and midwives, licenses qualified doctors and dentists, and maintains bacteriological laboratories. Palestine is nearly the size of Wales or Belgium, but it has only 800,000 [1] people, and barely a quarter of the cultivable land is regularly tilled. Almost every tree was cut down during the war, though some six millions of timber and fruit trees have been planted since.

[1] The population of 800,000 contains some 600,000 Moslems, 110,000 Jews and 75,000 Christians.

The country already grows 1,800 tons of tobacco a year, and exports oranges to the amount of £325,000 and wine to a total of £125,000. The Government, indeed, had to advance half a million of money to the war-stricken farmers, the greater part of which was promptly repaid. The revenue shows a small excess over expenditure, but it has been necessary to postpone any great irrigation or harbour works, much as they are needed. When the Jordan electric power works are completed, there will be a supply of 300,000 horse power available ; meanwhile temporary electric power and lighting stations have been formed at Jerusalem, Haifa, Jaffa and Tiberias. The potash contents of the Dead Sea water, 1,089 grains to the gallon, are likely to be utilised commercially before long, but minerals generally are deficient. Native agriculture and the cattle are wretched, apart from the 100 Zionist villages. The Jewish immigration, by the way, seems to be slackening, and the popular feeling against it to be subsiding. However, the Zionist movement has been a great benefit. They have expended some six millions of money in the country, started manufactures, reclaimed a large area of land, and by their example stimulated the industry of the natives. The British garrison is reduced to one company of armoured cars, one squadron of aeroplanes, and 450 British gendarmes.

Tell Gemmi lies nine miles south of Gaza, and is a grim mound in the rolling sandy plains. One side of it is a perpendicular cliff rising out of a huge dry wady, 250 feet broad and 30 to 50 feet deep. From the top we could see on the east the hills of Beersheba and Hebron, 30 or 40 miles away. On the north was a tiny glimpse of the blue Mediterranean. In the southern desert we could generally see an amusing mirage, showing a bay of water with houses and piers around it, where nothing but sand really existed. A very real and ancient camel track crossed the plain, " going down into Egypt," on which I counted 140 camels in line one day tramping along. At another time, as there was a famine in the land, we saw, day after day, Bedawyn tramping north, each family with its goods on a camel or donkey, and their half-starved sheep and goats trotting beside them. Here and there, across the plains, were little heaps of British barbed wire and trenches, an occasional Bedawy tent or hut, or solitary Arabs, each with a camel ploughing the ground for a very doubtful crop. On the west, but far out of sight, lay the village of Deir el Belah in its palm grove, the site of the Tommies' "Dear old Bella" hospital, and also of a fortress destroyed by our King Richard I.

Sir Flinders Petrie was excavating this mound, as it was the site of the Philistine city of Gerar, and probably a prehistoric camp. Here, as he tells us,[1] the Philistines kept a viceroy with the title of Abimelech to collect corn and export it to their homeland, Crete. Here Abraham and Isaac resorted, and some jealousy arose about the wells and corn which their tribe required, till they moved away to Beersheba. It was also a great border fortress, the scene of many Assyrian and Egyptian struggles. Indeed, Palestine has always been the Flanders of the near East from Sumerian times down to Napoleon and Allenby.

On digging into the level hill-top, strewn with endless fragments of pottery, one soon came down on great buildings, Persian granaries, Egyptian and Assyrian fortresses built of sun-dried bricks and littered with the weapons and scarabs, the idols and the cooking ovens of people who lived here from 1500 B.C. to 450 B.C. In the small section of one town-level excavated before I left, I counted about 28 chambers, some of them 30 feet by 15 feet, with walls still 12 or 15 feet high. There were, in fact, six towns, one built on the ruins of another, and below them the remains of camps of the Bronze and Neolithic periods. The date of each town could be approximately fixed from the things

[1] See *Ancient Egypt*, 1927, Parts I. and II. Macmillan & Co.

found in it. Thus immense quantities of pottery had to be sorted, drawn to scale and classified as Egyptian, native, Ægean, Cypriot or Cretan, of such and such a period. Coins, seals, a hundred and ninety weights for traders, bronze and iron lances and arrow heads, eighty flint sickles, an engraved Assyrian cylinder of lapis lazuli, a beautiful gold frontlet, nine gold earrings of about 1200 B.C., necklaces of carnelian agate and crystal, and various pottery figures were turned up to the delight of the diggers, who got a reward for each authentic find.

At the foot of the hill lay vestiges of a Roman town with cisterns, mosaic pavements, tombs, and the usual mounds of pottery. Someone has said that the Romans seem to have spent most of their time in making and breaking up pottery, and indeed a mound of fragments 30 feet high makes one realise the huge consumption of it, in the days when it replaced our barrels, cans and boxes, as well as our crockery. In a radius of three miles were the remains of four towns, showing the great population under the Roman peace, where now only a few Bedawyn get a scanty living. Whether under British rule it can once more be made a fertile country, by rebuilding the irrigation dams and by afforestation, remains to be seen. They have in this southern area 12·5 inches of rain, but it runs off at once, leaving the ground fissured in places very deeply, just like a photograph of the moon's surface.

For the greater part of our time our only water supply was a well two miles away in the desert, and the springs at Gaza nine miles distant. Camels had to be kept going bringing water from one place or the other, in great pottery jars, every day. Besides a few skilled Egyptians, Sir Flinders employed Bedawy men and boys, whose number gradually amounted to 370. Every Arab taken on as a digger required three children who carried off the earth in baskets, on their heads, and threw it down a shoot. What merry little imps these boys were! Their great delight was to push one of their fellows over the shoot, if not watched. Down he would roll a hundred feet or so, but always came up unhurt. Since men, girls and boys were all barelegged, cuts and sores were very common. It was impossible to put on dressings, except in the worst cases, so I went round while they were at work and painted them with iodine, or methylated spirit with a little biniodide. The patients cried out *kuwaiyis* (beautiful), especially if it smarted, and the results were excellent. One day some boys had a scrimmage, stones flew, and five or six cut scalps had to be dressed and sewn up. After work-hours we had a sick parade for serious cases, but the general health was good. Eye troubles were important and numerous. There were a few cases of subacute rheumatism, pleurisy and tonsillitis, and one or two of tuberculosis. Impetigo of the scalp was common, the home treatment for which was a plaster of sheep droppings. Curiously enough we had a slight outbreak of mumps. The cases were sent off at once, and duly notified to the Health Office at Gaza, on a trilingual form printed in Arabic, English, and Hebrew, as all the public notices and time-tables are made out. There were no snake bites, and only three or four cases due to scorpion stings, which returned to work in half a day or so, after incision and soaking in permanganate. Fortunately we had no malaria or sandfly fever, and there was little or no syphilis, though it was common in some districts near. The men all wore Arab costume, which is absurdly like a university gown, with a long shirt beneath, and a fringed towel tied round the head, but very effective and striking. The women and girls were always in black, closely veiled, and having great strings of coins hanging between their eyes. Sheiks and other well-to-do people would come round, laden with rifles and swords, on gorgeously caparisoned horses, but our men usually kept their weapons at home, or out of sight. Our watchman, or guardian appointed by the tribe, carried, indeed, a huge sabre, and was an expert slinger, by the way, like his ancestors. The Government had been powerful enough to stop all raids and tribal wars for the last year or two in the south, and it really was wonderfully peaceful and safe. One could wander about by day or night without risk or incivility. Once or twice a week mounted police patrols came round to ask whether we were all right. Every week, too, our camel went to Gaza to bring us the welcome post-bag, vegetables and fresh meat to add to our tinned foods. One was struck with the docility of the animals. Horses and donkeys rarely had bits, whips were little used, sheep of course followed the shepherd like dogs, and few, if any, of the beasts were castrated. The camels had views of their own about a proper load, but toiled along or knelt down at command, without blows as a rule.

One night I was invited to an entertainment, and went with a friend to an appointed spot near some tents. The moon and stars shone dimly, and we had a ceremonious welcome when we met our Bedawy hosts, who spread rugs for us on the sand and regaled us with tiny cups of sweet tea. Soon there were fifty men assembled, who formed a line, swaying to and fro, and chanting songs with wild refrains, sometimes advancing and sometimes retreating. This line of weird figures, some in their cloaks and some in pure white, ceaselessly singing and posturing, made a strange picture under the stars, and behind us was the great dark mound of Tell Gemmi. We might have been looking at some mystic rites of their Philistine ancestors ; but no, it was only a genial social " Fantasia " of poor Arabs who had been toiling hard all day. How they found the energy to dance and sing for three or four hours on their native sands astounded me. After a while one or two of the men went to the tents and fetched three women in their black robes wearing the long shawl which reached from head to foot. One of them, holding up her shawl, took a sword and waved it vigorously above her head, as she glided through a gentle dance in front of the line of singers, who alternately advanced and retreated before her. Another song seemed to imitate the growling of wild beasts, which was given most realistically. Here the woman appeared to be attacked by the baying animals, but drove them off by her sword. Other scenes followed, but no musical instruments accompanied the singers, though frequent rounds of hand clapping marked the cadences. The endurance of the men was shown later on in the Ramadan fast, when, in spite of dusty work, not a drop of water or food was taken from dawn till sunset. It was a fine performance of a hard duty, but the hours of labour had to be altered to meet their physical exhaustion. The people in this district are a mixed race ; most of them had black hair and eyes with good features and well-filled skulls, but there was at least one family with flaxen hair ; also a very few negroes. The Arabs in their bearing and habits are not unlike our Highlanders of 1745, and have similar failings, but they are proud fighting men, dignified and courteous to friends, and live the hardest of lives.

One day a sandstorm began and darkened the sky. Our clothes, books and bedding were soon full of the hateful dust. A hurricane of wind, hail and rain followed. The rattle of the hail on our iron roofs, hour after hour, was a thing to be remembered. During the night I was called out to attend to a student who had fainted, after running out in the storm to get help to replace a roof, which had been blown off. Two or three days of this downpour caused a flood around us, and turned our wady into a fine river, but in a week or so the sandy plains were dry again and the water all lost. In general, however, the climate in the winter months was perfect—daily sunshine and blue skies, and a temperature in the afternoon of 70° to 75°. Swallows and larks flew about the plain, and kites, kestrels and pigeons around our cliff; brown lizards played in the sun on every bank, while at night the jackals and Arab dogs called to each other under the brilliant stars and moon. At Gaza and Deir el Belah were palms and gardens of spinach, tomatoes, oranges, cucumbers and huge cauliflowers. Excellent fish, too, were caught in the sea. In a few years there will probably be fine watering places and good hotels on the Palestine coast, for the climate seems much better than that of the Riviera, and the sea is of the most exquisite blue imaginable. Indeed, Haifa and Beirut in French Syria are now charming places to winter in, and the Italians have built a huge hotel and casino at Rhodes. At Beirut at the foot of the Lebanon mountains there are good schools, and a wonderful American University, richly endowed and with fine buildings. It has a great medical school and printing press, and trains most of the doctors, engineers, civil servants and nurses for Palestine, Syria, the Sudan, Irak and the countries around. At Gaza one of the high school boys showed me a beautiful Arabic edition of Breasted's ancient history, printed at Beirut, which had been given to him.

The scenery of Syria among the Lebanon mountains, with the snow-clad Hermon, is magnificent; but

Palestine consists of a bare rocky table-land, with low-lying coastal plains on the west, which, for a few miles at Esdraelon only, spread right across the country and divide the table-land into two. On the south the low plains form a sandy desert down to Egypt, and on the east is the strange depression of the Jordan valley, which at Jericho is 1,500 feet below sea level. As Jerusalem is 2,500 feet above the sea, the change of climate is marked when one motors down the 21 miles of excellent road between them.

Jerusalem, except as to its modern suburbs, is dingy, old and crowded within high mediæval walls, a maze of lanes and arcades ; but idealised by the remembrance of the endless procession of men and women, Christian, Jewish and Moslem, who have come there, as pilgrims, to worship, from every corner of the world.

Archæologically the discoveries of authentic historical sites almost rival those of the Roman Forum. While I was there they were opening up a great forgotten wall commenced by Agrippa in A.D. 50, and finding near it some of the stone shot probably hurled by Titus in the siege of A.D. 70.

The finest building, of course, is the Mosque of Omar, standing on the site of the temples of Solomon and Herod. Then, too, there is the huge church of the Holy Sepulchre, a noble and impressive pile, but old and faded after 1,600 years of alternate existence, destruction, and repairs. In front of the doors lies the curiously preserved tombstone of Sir Philip Daubeny, a name well known in Bristol. A great charm of the church, to my mind, is that one enters it freely, and walks about at one's will, without fees or beggars. The trend of recent criticism, by the way, is strongly against the rival or Gordon site, outside the Damascus gate. As I am not writing a guide book, I must pass over the many interesting and authentic places, such as Bethany, Kedron and Bethlehem, which are easily visited.

At eight o'clock one morning I took my seat in a public motor-car, and in five or six hours reached Tiberias, a hundred miles north, passing through Shechem Nazareth and Cana. The road was in perfect order and ran along the table-land, then through fertile valleys and the wide plain of Esdraelon, dotted with Zionist villages, " Balfouria " and others, then up again among the hills of Galilee, till we saw far below us the blue waters of the lake embosomed in treeless but green hills. A few fishing-boats sailed about it, but except for Tiberias there was hardly a house or village to be seen. Close to Tiberias, indeed, are hot thermal springs where baths are being erected, and a charming Scottish mission hospital, in grounds full of shrubs and flowers, lies near the lake. As this is 680 feet below sea-level, the air is decidedly warm. The narrow old streets with many shops cluster round a good hotel and an excellent Franciscan rest-house. Some three miles away on the north are caves, where a Neanderthal skull was recently found. Except for a hospice or two, the sites of Bethsaida and Capernaum are a wilderness, but the ruins of the celebrated synagogue have been disinterred, and its white stone blocks, covered with sculptured leaves and flowers, lie on the ground like a child's box of bricks. Some of the columns have been re-erected, and it is quite easy to make out the plan of the building, an oblong, 75 feet long, divided by pillars into a nave and aisles. The richness of the decoration is striking, the star of David among vines and pomegranates being a prominent emblem.

Another motor-car took us on to Damascus, 85 miles, crossing the upper Jordan above the lake, where the French and English customs officers overhauled us, one on each side of the bridge. A little north of it is a swamp, where the papyrus still grows. It has disappeared everywhere north of Nubia, except at this spot and in the Cairo Zoological Gardens. We soon saw a different sight as we crossed the elevated plains beyond, viz. the white slopes of the snow-clad Hermon, and after another long spin the buildings of Damascus came in view, backed by a brown bare mountain ridge, and facing a plain full of trees and gardens. Through them and through the town itself tumble the rapid waters of the river Abana, reminding one of Innsbrück or Salzburg. The town has been stormed and sacked at short intervals from the dawn of history, and two years ago the French and the rebels contrived to batter down part of it again. An electric tram took one through a mile of ruins in one section.

It was fascinating to see the passenger cars come in from Baghdad, 540 miles away across the desert, which they do in twenty-four to thirty hours. The Damascus bazaars, especially that of the goldsmiths, and Nassam's huge manufactory of inlaid work and brass goods too, are of endless interest, and if one has the good fortune to stay, as I did, in a private house with a central court full of fountains and flowers, one realises what a charming residence it is in times of peace.

Once more, taking a motor, instead of the train, we went to Beirut, crossing the Antilebanon, then among the vines and mulberries of the rich plain of Caelo-Syria, and mounting the Lebanon range, where the road for miles ran among great snow-drifts, till at last we saw the blue sea in front of us, and began a long winding descent to the coast among olives and pines.

From Beirut an Italian steamer took us to Cyprus, passing the river of Adonis, and Tripoli, then along the coast of Asia Minor to Rhodes, Cos, and the Dardanelles.

The extraordinary beauty of the route was clouded by memories of the terrible events at Gallipoli and Smyrna, and the ghastly loss of life at both places. At Smyrna one of our passengers, who had landed to see the ruins, got arrested for taking a photograph, but succeeded in getting back. We spent some hours at Constantinople as well as at Athens and Naples, squeezed through the lofty cliffs of the Corinth canal, and reached Marseilles in good time.

